# Grafted Human Embryonic Progenitors Expressing Neurogenin-2 Stimulate Axonal Sprouting and Improve Motor Recovery after Severe Spinal Cord Injury

**DOI:** 10.1371/journal.pone.0015914

**Published:** 2010-12-30

**Authors:** Florence E. Perrin, Guillaume Boniface, Che Serguera, Nicolas Lonjon, Angeline Serre, Monica Prieto, Jacques Mallet, Alain Privat

**Affiliations:** 1 Neuroscience Department, University of the Basque Country UPV/EHU, IKERBASQUE Basque Foundation for Science, Bilbao, Spain; 2 INSERM U583, Institute for Neurosciences of Montpellier, Pathophysiology and therapy of sensory and motor deficits, Saint Eloi hospital, Montpellier, France; 3 Department of Neurosurgery, Gui de Chauliac Hospital, Montpellier, France; 4 Biotechnology and Biotherapy, Centre de Recherche de l'Institut du Cerveau et de la Moelle Epiniere, Centre National de la Recherche Scientifique (CNRS) UMR 7225, Institut National de la Santé et de la Recherche Médicale (INSERM) UMRS 975, Université Pierre et Marie Curie (UPMC) - Hôpital de la Pitié Salpêtrière, Paris, France; 5 MIRCen, CEA/INSERM, Modélisation des Biothérapies, Fontenay-aux-Roses, France; 6 Neureva Inc.-INM, Saint Eloi hospital, Montpellier, France; National Institute on Aging Intramural Research Program, United States of America

## Abstract

**Background:**

Spinal cord injury (SCI) is a widely spread pathology with currently no effective treatment for any symptom. Regenerative medicine through cell transplantation is a very attractive strategy and may be used in different non-exclusive ways to promote functional recovery. We investigated functional and structural outcomes after grafting human embryonic neural progenitors (hENPs) in spinal cord-lesioned rats.

**Methods and Principal Findings:**

With the objective of translation to clinics we have chosen a paradigm of delayed grafting, i.e., one week after lesion, in a severe model of spinal cord compression in adult rats. hENPs were either naïve or engineered to express Neurogenin 2 (Ngn2). Moreover, we have compared integrating and non-integrating lentiviral vectors, since the latter present reduced risks of insertional mutagenesis. We show that transplantation of hENPs transduced to express Ngn2 fully restore weight support and improve functional motor recovery after severe spinal cord compression at thoracic level. This was correlated with partial restoration of serotonin innervations at lumbar level, and translocation of 5HT1A receptors to the plasma membrane of motoneurons. Since hENPs were not detectable 4 weeks after grafting, transitory expression of Ngn2 appears sufficient to achieve motor recovery and to permit axonal regeneration. Importantly, we also demonstrate that transplantation of naïve hENPs is detrimental to functional recovery.

**Conclusions and Significance:**

Transplantation and short-term survival of Ngn2-expressing hENPs restore weight support after SCI and partially restore serotonin fibers density and 5HT1A receptor pattern caudal to the lesion. Moreover, grafting of naïve-hENPs was found to worsen the outcome versus injured only animals, thus pointing to the possible detrimental effect of stem cell-based therapy *per se* in SCI. This is of major importance given the increasing number of clinical trials involving cell grafting developed for SCI patients.

## Introduction

Spinal cord injury (SCI) is a devastating pathology with currently no effective treatment of any symptom, that often leads to permanent loss of motor, sensory and autonomic functions (for review see [1], [2]). In SCI, lack of spontaneous axonal regeneration, and the correlative absence of functional recovery, is not primarily due to an endogenous inability of axon to re-grow [3] but is impeded by a combination of inhibitory factors, including astrocyte scarring [4]. Many studies have investigated the effects of grafting a variety of cells in several animal models of spinal cord injury ([5], [6], for review see [7], [8], [9], [10], [11]) and have reported beneficial but also severe detrimental side effects [12]. Cell transplantation may be used in different non-exclusive ways to promote axonal regeneration and functional recovery after spinal traumatism: (1) to bring permissive molecules and/or trophic factors at the lesion level to enhance the regenerative capacity; (2) to provide a scaffold for the regeneration of severed axons; (3) to replace lost cells. Thus, any strategy aiming at repair of a damaged spinal cord must be elaborated so as to fulfill one or several of the above listed objectives. Human embryonic neural progenitors (hENPs) are predetermined to differentiate into neural lineages (for review see [11]) but gene modification prior to their transplantation may enhance their survival, control their differentiation and deliver transgene products. In order to reconstruct the neuronal circuitry damaged by the lesion, transplantation of genetically modified cells expressing proneural factors is thus an appealing therapeutic strategy. Neurogenin-2 (Ngn2) is an attractive candidate since it is a proneural gene involved in neuronal differentiation and subtype specification in various regions of the nervous system but which also inhibits astrocytic differentiation [13], [14].

With the aim of translation to the clinic, we explored the transplantation potential of hENPs either naïve, or engineered to express Ngn2 in a severe model of spinal cord compression in adult rats. We report here that hENPs expressing Ngn2 induce a functional motor recovery associated with a partial restoration of serotonin fibers and appropriate location of serotonin receptors below the lesion site. Moreover, we evidence that hENPs transduced with a non-integrating lentiviral vector to express Ngn2 induce similar recovery than those transduced with an integrating vector. Conversely, transplantation of naïve hENPs led to worsening in functional outcome.

## Results

### Ngn2-hENPs improved functional motor recovery whereas naïve hENPs worsened it

Adult rats were submitted to severe spinal cord compressive injury at thoracic level (T8–T9) [23]. One week later (sub-acute time window), they were transplanted with hENPs. We then assessed their motor, sensory and reflex functions over one month, before sacrifice. Behavioral recovery was compared in four groups of animals: 1) injured-only animals, 2) injured animals grafted with naïve hENPs transduced with a GFP expressing lentiviral vector, 3) animals grafted with hENPs genetically modified to express Ngn2 with an integrating (Trip-PGK-Ngn2) vector, 4) animals grafted with a non-integrating (Ni-Trip-PGK-Ngn2) lentiviral vector [15].

**Figure 1:**
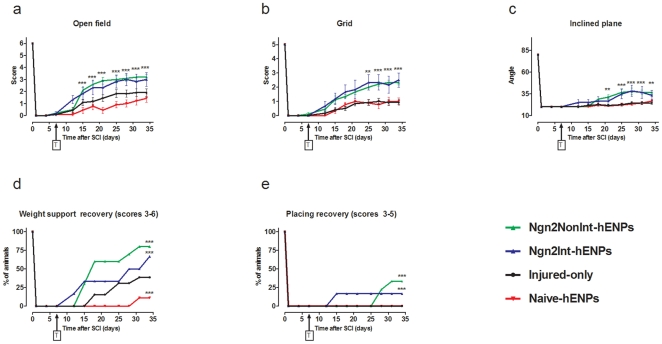
Transplantation of hENPs genetically modified to express neurogenin-2 improved functional motor recovery whereas naïve-hENPs grafting worsened it. (**A&D**) Open field test: walking on the horizontal plane. (**A**) Open field test before and during 35 days post-spinal compression (28 days post-transplantation (T)) for evaluation of gross motor function. From 7 days after transplantation, rats grafted with both non-integrating and integrating-Ngn2-hENPs presented a higher motor recovery than injured-only and naïve-hENPs grafted animals. No difference was seen between both Ngn2 groups. Rats transplanted with naïve-hENPs presented a lower recovery than injured-only animals. (**D**) A higher percentage of animals transplanted with Ngn2-hENPs presented a functional motor recovery that correspond to weight support (score 3 to 6). Transplantation with naïve-hENPs worsened motor outcomes as compared to injured-only rats. Scores: 0 corresponds to no hind limb movement and no weight bearing; 3 to weight support and 6 to normal walking. (**B&E**) Grid navigation test for evaluation of fine motor coordination over a 50-cm horizontal runaway grid. Scores grid: 0 correspond to hind limb drag without foot placement and 5 to normal walking over the grid with toes gripping wire. (**B**) From 21 days after transplantation, rats grafted with both non-integrating and integrating-Ngn2-hENPs presented a similar higher motor recovery than injured-only and naïve-hENPs grafted animals. (**E**) A higher percentage of animals transplanted with Ngn2-hENPs presented a fine motor recovery that correspond to griping of the grid (score 3 to 5). (**C**) From 21 days after transplantation, rats grafted with both non-integrating and integrating-Ngn2-hENPs presented a similar higher performance on the inclined plan test than injured-only and naïve-hENPs grafted animals. T =  transplantation. Statistical analysis: (**A–C**) two-ways ANOVA analysis followed by Bonferroni's multiple comparison test (**: p<0.01; ***: p<0.001). (**D&E**) Fisher's exact test on the percentage of animals reaching a given score one month after grafting was used (**P<0.01, ***P<0.001). Injured-only rats (control group 1, n = 11); naïve-hENPs (control group 2, n = 9); Ngn2-non integrating (n = 10) and Ngn2-integrating (n = 6).

As early as 8 and 14 days after transplantation, rats transplanted with integrating and non-integrating Ngn2-hENP presented a better gross motor recovery (open field, Figure 1A, inclined plan, Figure 1C) than naïve-hENPs-grafted and injured-only animals. Ngn2-hENPs grafted groups reached an efficient functional score corresponding to a weight support capacity (score of 3; open field test). One month after transplantation the percentage of animals that were able to support their weight (score 3–6, open field) in both Ngn2-hENPs transplanted groups was significantly higher than in naïve-hENPs-grafted and injured-only groups (Figure 1D). Importantly, animals grafted with naïve-hENPs had a significantly worse gross motor recovery than injured-only rats (Figure 1A&D).

Eighteen days after transplantation, fine motor function evaluated by a grid-navigation test was also significantly better for Ngn2-hENPs grafted rats that presented a correct hind limb placement (grid, score of 2) or even griping on the grid (score of 3) (Figure 1B&E). This improvement was noticeable since 7 to 10 days post grafting. Moreover, the percentage of Ngn2-hENPs grafted animals that reached a grid score ≥3 was significantly higher when cells had been transduced with a non-integrating vector than with an integrating vector (Figure 1E). For all other tests, animals grafted with persistent (integrating vector) and transient (non-integrating vector) Ngn2 expression recovered similarly (Figure 1). No differences were observed between all groups for sensory, reflex and autonomic functions, with the exception of bladder control which was significantly improved in integrating-Ngn2-hENPs grafted rats (Supplementary Figure S1).

### Lesion extension is not modified by Ngn2-hENPs

**Figure 2:**
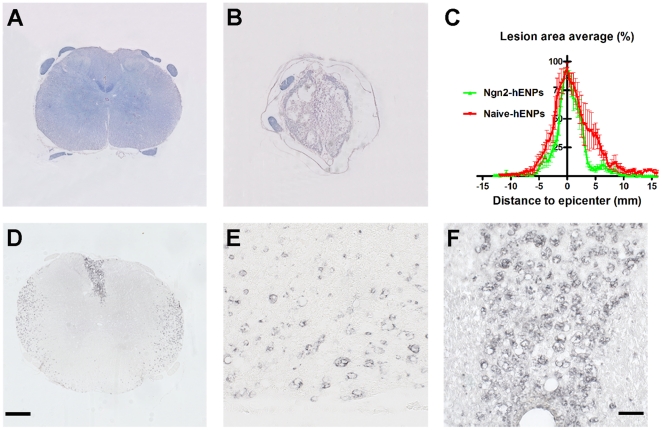
Histological analysis of the injured spinal cord. (**A–C**) Grafting of non-integrating-Ngn2-hENPs did not modify lesion extension after spinal cord compression. (**A&B**) Photomicrographs of Luxol fast blue stained sections of an injured spinal cord grafted with naïve-hENPs. Transverse sections of the spinal cord taken at different levels: (**A**) rostral to the lesion (**B**) Epicenter of the lesion. (**C**) Percentage of lesion area at the epicenter 5 weeks after spinal cord injury in non-integrating-Ngn2-hENPs and naïve-hENPs transplanted rats. At the epicenter both groups presented a lesion area of 90% and a rostro caudal extension of 15 mm. (**D–F**) ED1 staining revealed massive macrophage activation at the injury site and at grafting levels. At transplantation levels macrophages were localized at the injection point (**D&F**) and in the peripheral white matter (**2D&E**). Photographs had been taken in a non-integrating-Ngn2-hENPs grafted rats but macrophage activation was similar in all transplanted groups. Scale bars: (**A, B&D**) 200 µm and (**E&F**) 50 µm. Naïve-hENPs (n = 9); Ngn2-non integrating (n = 10) and Ngn2-integrating (n = 6).

In a complementary series of experiments intended to compare lesion extension in naïve and non-integrating-Ngn2-hENPs grafted rats, we carried out histological analysis of the injured spinal cords (Figure 2A–C). We evaluated the lesion area on a large segment (3-cm corresponding to 3 metamers) of the spinal cord centered on the lesion that included the 3 grafting points. One month after transplantation, there was no difference in the extension of the injury at the epicenter (non-integrating-Ngn2-hENPs: 93.1+/−1.5% and naïve-hENPs: 89.9+/−9.6). Means of areas under the curves, corresponding to the total amount of damaged tissues (that is, volumetric analysis according to the Cavalieri principle [16]), were not significantly different in both groups (440.5+/−36.2 and 659.5+/−93.6 (arbitrary units) in the non-integrating-Ngn2-hENPs and naïve-hENPs grafted groups) (Figure 2C). Thus, these experiments showed that Ngn2-hENPs grafting did not reduce lesion extension and indicated that only 10% of preserved tissues at the lesion site was sufficient to permit improvement up to full weight support.

### hENPs were not present one month after grafting

One month after transplantation the fate of grafted hENPs was analyzed on histological coronal sections through a 3 cm spinal cord segment centered on the lesion site. Since all grafted cells were engineered to express GFP, we first searched for green fluorescent cells in the transplanted area. Because major auto fluorescence was observed in all groups, including that of injured-only rats, we further used peroxidase-conjugated secondary antibodies to detect GFP or other specific markers. In all grafted animal groups (naïve-hENPs, non-integrating-Ngn2-hENPs and integrating-Ngn2-hENPs) no hENPs were ever detected using GFP immunostaining. Since we could not exclude that GFP expression decreased 4 weeks after transplantation and since less of 100% of the grafted cells were actually GFP positive, we searched for the presence of the antigen HuNu to identify human cells; again, no positive cells were ever detected. As auto fluorescence is a known property of macrophages [17], [18], [19] probably due endogenous flavoproteins [20], [21] and since auto fluorescent cells were morphologically macrophage-like cells we then searched for the presence of macrophages. ED1 staining revealed massive macrophage activation at the injury site and at grafting points (Figure 2D–F) that correspond to auto fluorescent cells. At the epicenter of the lesion macrophages were present all over the spinal cord sections, whereas at transplantation levels macrophages were localized at the injection points (Figure 2D&F) but also in the peripheral white matter (Figure S2D&E). Macrophage activation was similar in all-3 groups of grafted rats. In injured-only animals, ED1 staining was similarly present at the epicenter.

### Ngn2-hENP partially restored serotonin and 5HT1A receptors expression

**Figure 3:**
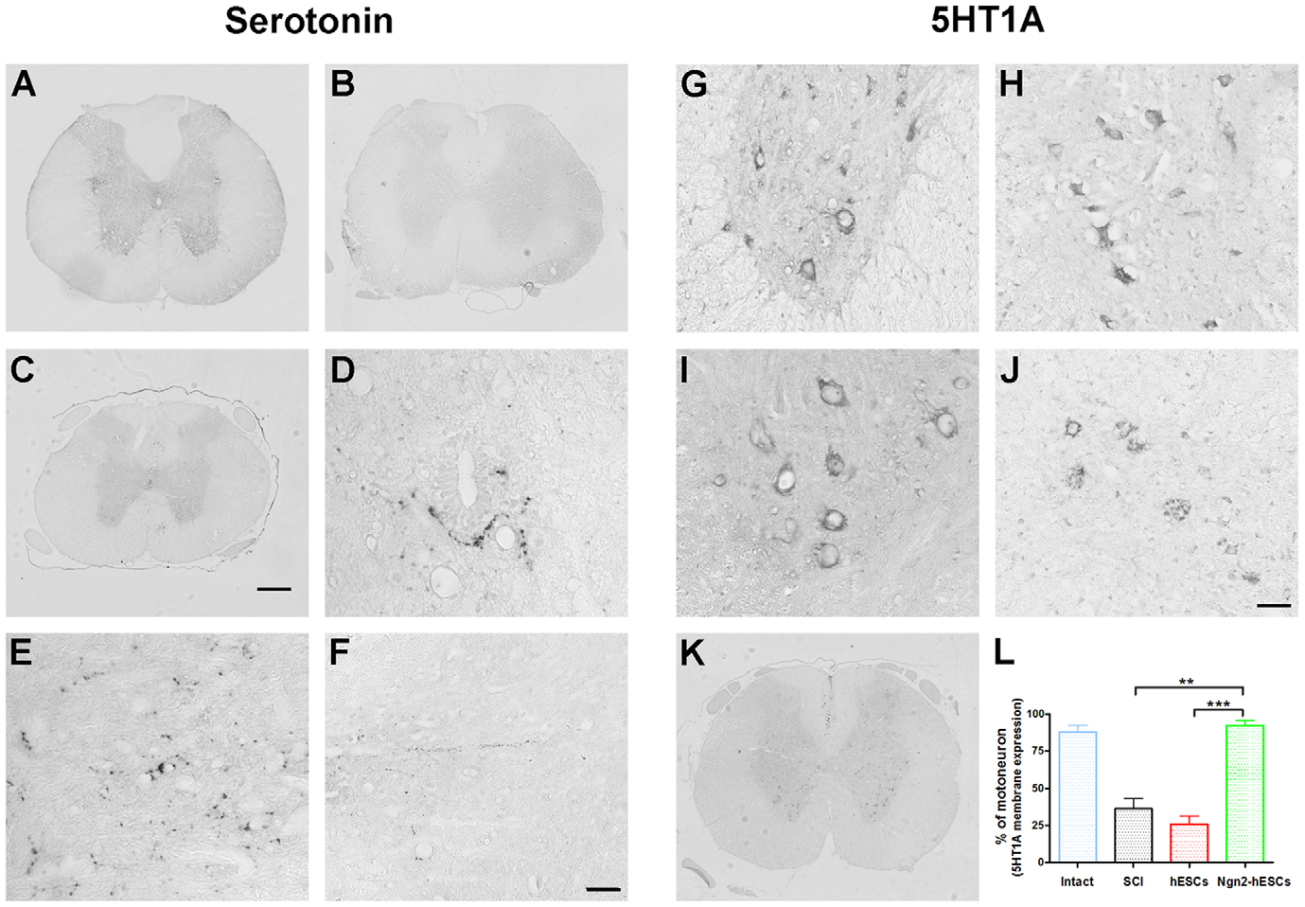
Transplantation of hENPs genetically modified to express Ngn2 partially restored serotonergic fibbers density and distribution of 5HT1A receptors caudal to the lesion. (**A–F**) Serotonin expression pattern 1 mm below the injury site 4 weeks after transplantation. (**A**) Serotonergic fibbers (5HT) are located in the ventral horn of uninjured rats. (**B**) 5HT expression below the lesion site is dramatically reduced in naïve-hENPs-grafted rats. (**C–F**) In rat transplanted with non-integrating-Ngn2-hENPs 5HT expression is partially restored below the lesion. Thin 5HT positive fibbers are present over the ventral horn in the vicinity of motoneurons (**C&E**) they spread toward the central canal (**D**) and the white matter (**F**). (**G–K**) Serotonin receptor type 1A (5HT1A) expression pattern 1 mm below the injury site 4 weeks after transplantation. (**G&l**) In intact spinal cord almost 90% of motoneurons have a membrane-bound localization of 5HT1A. (**H&L**) Five weeks after compression, 36% of motoneurons of the injured-only group have a 5HT1A membrane-bound localization as compared to 92% in the non-integrating-Ngn2-hENPs transplanted group (**I, K&L**). (**J&L**) 25% of motoneurons in rats grafted with naïve-hENPs expressed 5HT1A at the membrane. (**L**) Quantification of the motoneurons that express 5HT1A in a membrane location. One-way ANOVA analysis followed by Bonferroni's multiple comparison test was used for analysis of the location of 5HT1A receptors; (**: p<0.01; ***: p<0.001). Scale bars: (**A, B, C&K**) 200 µm and (**D–J**) 50 µm. Uninjured animals (n = 6), injured-only rats (n = 11); Naïve-hENPs (n = 9) and Ngn2-non integrating (n = 10).

The raphe-spinal serotonergic system plays a major role in locomotion and is particularly affected after SCI [22], [23]. To get further insight into the mechanism of functional improvement, we characterized four weeks after transplantation serotonergic fibers (5HT), on a spinal cord segment located 1 mm below the lesion site in injured-only and grafted animals. In intact spinal cord, serotonergic fibers were present in the ventral horn surrounding their alpha-motoneuron targets and in the intermediolateral cell column (IML) (Figure 3A). Five weeks after spinal cord compression, 5HT expression was dramatically reduced in injured-only and naïve-hENPs-grafted groups (Figure 3B). Conversely, both Ngn2-hENPs transplanted-groups (integrating and non-integrating) presented a substantial restoration of serotonergic profiles (Figure 3C–F). Numerous thin fibers were present over the ventral horn in the vicinity of alpha-motoneurons (Figure 3C&E). Serotonin expression appeared in long and varicose fibers in the IML and spread medially toward the central canal as well as laterally within the white matter (Figure 3D&F).

We then looked for the presence and cellular location of corresponding receptors. In the same spinal segment 88% of alpha-motoneurons presented a membrane-bound localization of the serotonin receptor type 1A (5HT1A) in intact spinal cord (Figure 3G&L) as compared to 36.4% five weeks after SCI for injured-only rats (Figure 3H&L). Four weeks after non-integrating-Ngn2-hENPs transplantation, sub-cellular localization of 5HT1A expression was entirely restored to intact pattern since 92.3% of alpha-motoneurons displayed 5HT1A expression at the membrane (Figure 3I, K and L). In the naïve-hENPs group, as in injured-only rats, only 25.8% of alpha-motoneurons expressed 5HT1A at the membrane, (Figure 3J and L).

Our results provide evidence that increased serotonergic fibers density caudal to the lesion in Ngn2-hENPs transplanted rat is associated with a presumed “active” distribution of 5HT1A receptors. These changes coincide with improved motor function observed in non-integrating-Ngn2-hENPs transplanted rats.

## Discussion

We have shown that sub-acute transplantation of Ngn2-hENPs in a model of severe SCI improves functional motor outcome. Recovery is associated, below the lesion site, with partial restoration of serotonin expression and translocation to the membrane of 5HT1A receptors in alpha-motoneurons. Moreover, we provide evidence that transplantation of naïve hENPs is detrimental to functional recovery.

We have designed our experiments with the objective of translation to the clinic. Indeed, spinal cord compression is a major component in most human injuries and most of these injuries are severe. Spinal balloon-compression injury in the adult rat is a well-characterized, reproducible and predictable SCI model; balloon inflation of 15 µl for 5 minutes in the vertebral canal at thoracic level 9 leads to severe spinal injury associated with complete paraplegia [24]. We have chosen a sub-acute time window (7 days) for grafting, since earlier grafting could interfere with spinal shock and edema whereas in the chronic phase detrimental presence of fibrotic tissues may reduce if not abolish expected benefits. Transplanted cells were of human origin and, as a tool for gene transfer, we have compared integrating and non-integrating lentiviral vectors, since the latter associate a high transducing efficiency with reduced risks of insertional mutagenesis [15]. Behavioral tests [24], [25], [26] were chosen to evaluate authentic functional recovery (weight support capability) rather than modification in limbs positioning that not always represent a functional improvement.

Others had shown that transplantation of rat adult neural stem cells in a rodent model of spinal weight-drop injury improved functional recovery but also increased neuropathic pain whereas previous cells transduction with neurogenin-2 prevented astrocytic differentiation and allodynia [12]. At variance, we did not observe differences between all groups (injured-only, naïve-hENPs and both hNgn2-hENPs) for sensory, reflex and autonomic functions. In our model, severe compression of the dorsal aspect of the spinal cord might initially produce major allodynia and/or hyperpathia phenomena [13] that would hinder further evaluation of any sensory worsening. Thus, our results can hardly be compared with those of Hofstetter et al. [12] since those authors used a different lesion model. Moreover, transplanted cells were of different origin, since adult rat neural stem cells were grafted [12] whereas we transplanted human embryonic neural progenitors.

Four weeks after grafting, hENPs were undetectable in the injured spinal cord. Thus, not only does a transient expression of Ngn2 suffice to achieve motor functional recovery but also functional benefits obviously do not result from replacement of lost cells. Motor recovery induced by transplanted cells appears thus to be indirect and is likely due to activation of endogenous preserved circuitry via trophic support supply and/or generation of favorable substrate for axonal growth as previously hypothesized ([27], (for reviews see [7], [28]). In this respect, worth noting is the fact that no more than 10% of spinal cord tissue was preserved at the level of the injury. A possible alternative mechanism could be through the protection of endogenous cells from further secondary damage, but this appears unlikely due to the delay of one week in between lesion and grafting.

The rapid recruitment of a large number of macrophages that we observe at the lesion site is a well-characterized event [29], [30] and it is likely that the presence of macrophages at injection sites results from trauma-induced inflammation due to spinal cord needle penetration and cell transplantation. Such inflammatory response may contribute to the loss of grafted cells that however contrasts with early and persistent improvement of motor functional recovery induced by transplantation of Ngn2-hENPs.

To conclude, transplantation of short term surviving hENPs that express Ngn2 restores weight support and grid walk after severe spinal cord compression. It corresponds with partial restoration of serotonin fibers density and of an “active” 5HT1A receptor pattern caudal to the lesion. Given the clear functional improvement observed in spinal cord injured-rat transplanted with human neural progenitors engineered to express Neurogenin-2, it is very important to elucidate the mechanisms of action that underlie this positive outcomes. This will be addressed in future studies that will be carried out at the histological and molecular levels. Moreover, since grafting of naïve-hENPs was found to worsen the outcome versus injured only animals, we call attention to the possible detrimental effect of stem cell-based therapy *per se* in SCI. This is of major importance given the increasing number of clinical trials of cell grafting developed for SCI patients.

## Materials and Methods

### Preparation of hENPs and viral transduction

Primary embryonic human neural progenitors (hENPs) were obtained from dissected telencephalic vesicles of 8-weeks-old aborted fetuses (legal date limit in the French legislation: 5–12 weeks) with the written consent of the fetus carrier, following the French (Law n° 2004–800 published in the “Journal Officiel”, 7^th^ of August 2004), the European legislations and the Declaration of Helsinki. The «Agence de Biomédecine», France, gave ethic approval for all steps of our study and for all participants. Abortions were performed in the Hospital Robert Debré, Paris, France and anonymity of the fetus carrier was preserved. Cells were amplified in culture as previously described [31], [32]. Briefly, progenitor cells were plated at a 6×10^4^ cells/cm^2^ density on plastic dishes coated with 0.25% gelatin and 10 µg/ml laminin and amplified in DMEN-F12 complemented with N2, B27 (all from Gibco, Carlsbad, USA), bFGF (10 ng/ml) and EGF (10 ng/ml) (both from Sigma Saint Louis, USA). Cells were passaged after 3–5 divisions using accutase (PAA, Pasching, Austria) for dissociation. For lentiviral vector transduction, dissociated neural stem cells were kept in the same media at a 10^6^ cells/ml density and incubated for 2 hours with 50 ng/p24 of vector stock per 1.2×10^5^ cells. We used four types of flap bearing HIV-1 derived lentiviral vectors [33], either expressing GFP or human neurogenin-2 cDNA under control of the house keeping promoter of the phosphoglycerate kinase promoter (PGK). Vectors either carried a wild type or a mutant integrase (D64V mutant) leading respectively to integrating and non-integrating phenotypes [15]. These vectors are named Trip-PGK-GFP and Trip-PGK-Ngn2 for integrating vectors and Ni-Trip-PGK-GFP and Ni-Trip-PGK-Ngn2 for non-integrating vectors. In both cases cells transduced to express Ngn2 were co-transduced with the respective (Trip or Ni-Trip) PGK-GFP vectors.

### Spinal cord compression and hENPs transplantation

Experimental procedures followed the European legislative, administrative, and statutory measures for animal experimentation (86/609/EEC). The study was approved by the “Direction des Services Vétérinaires de l'Hérault”, France (Alain Privat, authorization number 34118) and ratified by the “Préfecture de l'Hérault”, France. Every effort was made to minimize the number of animals and their suffering. We used 8 to 9 weeks of age female Sprague Dawley rats (220–240 g; n = 36) (Charles River, l'Arbresles, France). Surgeries were conducted under anesthesia with 1 L/min of O_2_ supply; anesthesia was induced by 4% isoflurane and maintained at 2.5% during spinal cord compression [24], [34]. Compression injury was carried out at thoracic level 9 (T9) similarly to that described previously [24], [26], [35], [36]. Briefly, a burr hole was drilled in the posterior arch of T10 vertebra to insert a 2-French Fogarty catheter (Edwards Life sciences, Horw, Switzerland) into the epidural space; the balloon was positioned at T9 level and water balloon inflation (15 µl) was made with a Hamilton syringe. The inflated balloon was left in place for 5 min. Seven days post-injury we grafted genetically engineered human embryonic neural progenitors (hENPs) in the spinal parenchyma. Over the course of the experiments we grafted primary hENPs issued from two series of cell preparation emanating from the same fetus. To ensure sufficient cell diffusion around the injury site, cells were injected in 3 points (lesion site, below and above); burr holes were drilled in posterior arches of T8 and T9 and T10 hole was re-opened). We injected a total of 90,000 cells (2 µl/site; 15,000 cells/µl) using a micro-electrode (80 µm of diameter) connected to a micro-injector (0.2 µl/min) and to a stereotactic micromanipulator. To diminish possible deleterious effects (such as spinal cord dissection) of transplantation that could lead to neurological worsening, cells were injected slowly (10 min/injection point) followed by a 5 minutes delay before needle withdrawing to avoid loss of transplanted cells resulting from suction phenomena. All cells were transduced to express either GFP protein only or in combination with Ngn2. For Ngn2 transduced cells we used either integrating or non-integrating lentiviral vectors. We thus had four animal groups: injured-only rats (control group 1, n = 11); GFP (control group 2, n = 9); Ngn2-non integrating (n = 10) and Ngn2-integrating (n = 6). Animals were excluded if they encountered at least one of the following exclusion criteria: unusual suffering, loss of weight exceeding 20% of initial measurement and insufficient primary lesion leading to a non-complete paraplegia before grafting. Percentages (less than 5%) of excluded animals were similar in all groups.

Animals were killed 35 days post injury with an overdose of pentobarbital and perfused transcardially with 4% paraformaldehyde in PBS. For histology, a 3 cm spinal cord segment (i.e. 3 metamers) centered on the lesion was dissected, post-fixed, cryoprotected (30% sucrose over night at 4°C) and embedded in Tissue-Tek OCT Compound (Sakura Finetek, Torrance, CA, USA).

### Pharmacological treatment

All rats received daily sub-cutaneous injection of Cyclosporin A (CsA) (Sandimmun, Novartis, Basel, Switzerland) 10 mg/kg. CsA injection started 2 days prior transplantation (5 days after lesion for injured-only rats) and until the end of the study (J35). Gentamycin antibiotic therapy was given by daily intramuscular injection (2 mg/kg) to avoid urinary tract infection for 15 days starting at lesion time [37].

### Behavioral monitoring

Before injury, and every third day during the whole study time, behavioral tests were carried out for motor, sensory and reflex functions as previously described [24], [25], [26]. Motor response was evaluated by 3 tests: open field walking and inclined plan for gross motricity and a grid-navigation test for fine motricity. Evaluation of sensory response consisted on pain and heat withdrawal for superficial function and proprioception for deep function. Reflexes were evaluated by hind limb withdrawal after manual extension and toes extension when the animal is picked up by the tail. Bladder function was assessed every day and manually emptied until subjects regained control. An independent experienced individual carried out all evaluations on a blind basis.

### Histology and immunohistochemistry

Lesion extension was analyzed on 12 µm-thick cryosections of a 3 cm spinal cord segment centered on the lesion site; one section each 360 µm was stained with luxol fast blue. Morphometric quantification of injured tissues was done on scanned images (Nano Zoomer Digital Pathology System, Hamamatsu, Japan) with MetaMorph software (MDS Analytical Technologies, Canada) as previously described [24], [26].

For immunohistochemistry, 12 µm sections of the same spinal segment were mounted on slides, washed in PBS (5 min), treated for 30 min in PBS containing lysine (20 mM, pH 7.4) and for 20 min in 3% H_2_O_2_). Tissue sections were then permeabilized and blocked for 30 min with PBS containing bovine serum albumin (BSA, 10%) and Triton X-100 (0.1%).

Rat polyclonal anti-ED1 (1∶500; Serotec, Oxford, UK), rabbit anti-5-hydroxytryptamine (1∶30000; Immunotech, Marseille, France), rabbit anti-5HT1A (1∶500; a gift from Dr. Michel Hamon INSERM U677 Neuropsychopharmacology Unit, Paris, France), mouse anti-GFP (1∶500; Abcam, Cambridge, UK) and mouse anti-HuNu (1∶250; Chemicon, Bilerica, USA) primary antibodies were used. Secondary peroxydase-conjugated antibodies (Immunotech, Marseille, France) were used at a 1∶500 dilution. The sections were scanned and analyzed (Nano Zoomer Digital Pathology System, Hamamatsu, Japan). The controls were done in the absence of the primary antibody and were negative in all cases.

### Statistical analyses

Behavioral analysis: two-ways ANOVA analysis followed by Bonferroni's multiple comparison test or Fisher's exact test on the percentage of animals rising a given score. T test was used for lesion extension at the epicenter and areas under the curves. One-way ANOVA analysis was used for analysis of the location of 5HT1A receptors; (*: P<0.05; **: p<0.01; ***: p<0.001).

## Supporting Information

Figure S1 **Transplantation of hENPs did not modify sensory outcomes but bladder control was improved in by integrating-Ngn2-hENPs grafting.** (**A**) Pain and (**B**) heat withdrawal was used to evaluate superficial sensory function. Scores range from normal response (0) to hyperalgesia (4). No difference was seen between groups, they were all similarly hyperalgesic. (**C**) Deep sensory function was evaluated by means of placement response of the hind limb (proprioception). (**D**) Reflexes were evaluated by hind limb withdrawal after manual extension; both groups presented similar deficits. Scores range from normal response (0) to hyper reaction (3). (**E**) Autonomic function corresponds to bladder control. Scores: no bladder control (0) and bladder control (1). (**A–E**) Statistical analysis: two-ways ANOVA analysis followed by Bonferroni's multiple comparison test. T: transplantation. Injured-only rats (control group 1, n = 11); naïve-hENPs (control group 2, n = 9); Ngn2-non integrating (n = 10) and Ngn2-integrating (n = 6).
(TIF)
