# Supplementary figures and images for: Grafted Human Embryonic Progenitors Expressing Neurogenin-2 Stimulate Axonal Sprouting and Improve Motor Recovery after Severe Spinal Cord Injury
Source: PLoS One. 2010 Dec 30;5(12):e15914. doi: 10.1371/journal.pone.0015914 (PMC3012721; doi:10.1371/journal.pone.0015914)

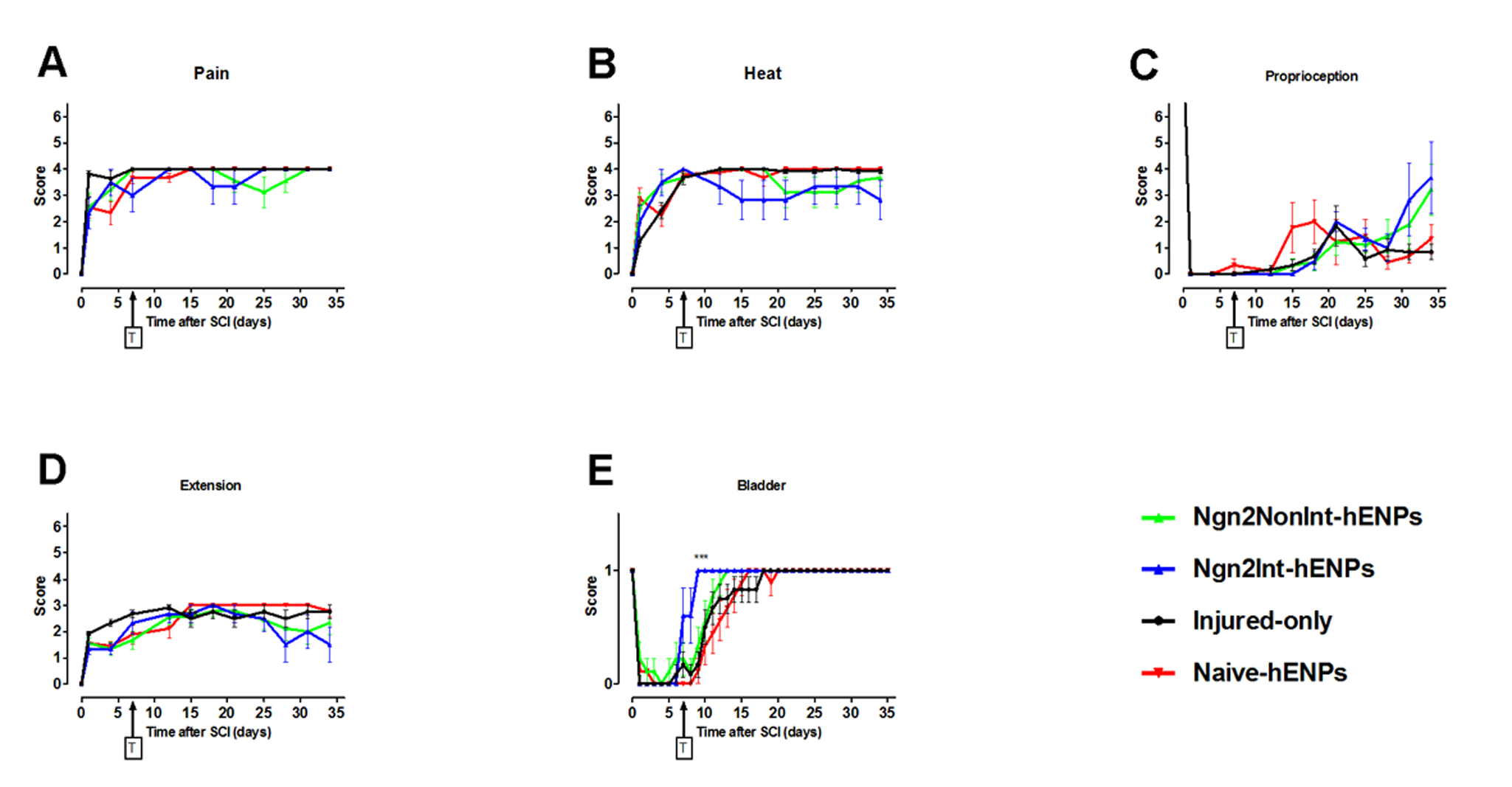

Supplement: Figure S1 — Transplantation of hENPs did not modify sensory outcomes but bladder control was improved in by integrating-Ngn2-hENPs grafting. (A) Pain and (B) heat withdrawal was used to evaluate superficial sensory function. Scores range from normal response (0) to hyperalgesia (4). No difference was seen between groups, they were all similarly hyperalgesic. (C) Deep sensory function was evaluated by means of placement response of the hind limb (proprioception). (D) Reflexes were evaluated by hind limb withdrawal after manual extension; both groups presented similar deficits. Scores range from normal response (0) to hyper reaction (3). (E) Autonomic function corresponds to bladder control. Scores: no bladder control (0) and bladder control (1). (A–E) Statistical analysis: two-ways ANOVA analysis followed by Bonferroni's multiple comparison test. T: transplantation. Injured-only rats (control group 1, n = 11); naïve-hENPs (control group 2, n = 9); Ngn2-non integrating (n = 10) and Ngn2-integrating (n = 6). (TIF) [file pone.0015914.s001.tif]
